# Transcriptional control of the MUC16 promoter facilitates follicle-stimulating hormone peptide-conjugated shRNA nanoparticle-mediated inhibition of ovarian carcinoma *in vivo*

**DOI:** 10.1080/10717544.2018.1451934

**Published:** 2018-03-15

**Authors:** Ming-Xing Zhang, Shan-Shan Hong, Qing-Qing Cai, Meng Zhang, Jun Chen, Xiao-Yan Zhang, Cong-Jian Xu

**Affiliations:** a Obstetrics and Gynecology Hospital, Fudan University, Shanghai, China;; b Department of Obstetrics and Gynecology of Shanghai Medical School, Fudan University, Shanghai, China;; c Shanghai Key Laboratory of Female Reproductive Endocrine Related Diseases, Shanghai, China;; d Department of Pharmaceutics, School of Pharmacy, Fudan University, Shanghai, China

**Keywords:** Ovarian carcinoma, MUC16, follicle-stimulating hormone, targeted therapy, nanoparticle

## Abstract

Ovarian cancer is the leading cause of cancer death among gynecological malignancies. The high mortality rate has not been significantly reduced despite advances in surgery and chemotherapy. Gene therapy shows therapeutic potential, but several key issues must be resolved before clinical application. To minimize toxicity in noncancerous tissues, tumor-specific ligands are conjugated to vectors to increase the selectivity of drug delivery. The expression pattern of follicle-stimulating hormone (FSH) receptor in normal and cancer tissues provides an opportunity for highly selective drug delivery in ovarian cancer. Furthermore, tumor-specific promoters can conditionally regulate therapeutic gene expression in tumor or normal tissues. The mucin 16 (MUC16) promoter might be a potential tool to drive ovarian cancer-localized gene expression since MUC16/CA125 is overexpressed in most ovarian carcinomas. Here, we screened the possible MUC16 promoter sequences and constructed MUC16 promoter-driven gro-α shRNA plasmid vectors. The vectors were specifically delivered into ovarian cancer cells via FSH peptide-conjugated nanoparticles. The predicted promoter sequence with TAAA repeats showed high transcriptional activity. The nanoparticle complex containing MUC16 promoter-driven gro-α shRNA and FSH peptides had the ability to decrease gro-α protein secretion in ovarian cancer cells and block tumor growth without obvious toxic effects in a nude mouse model bearing ovarian cancer. Our study provides a novel gene delivery system using a MUC16 promoter trigger and FSH peptide-mediated active targeting in ovarian cancer, and this system may be a promising strategy for specific genetic therapeutic delivery.

## Introduction

Ovarian cancer is the leading cause of cancer death among gynecological malignancies. The high mortality rate has not been significantly reduced despite advances in surgery and chemotherapy. Clinical trials of chemotherapy regimens, such as dose-dense weekly chemotherapy and single-agent nonplatinum treatment, have failed to improve survival outcomes (Suh et al., 2017). However, therapeutic strategies based on individual specific genetic abnormalities show potential clinical value. For example, a randomized, double-blind, phase-3 trial has shown that niraparib, a PARP inhibitor, can significantly improve progression-free survival among patients with platinum-sensitive, recurrent ovarian cancers (Mirza et al., 2016).

Similarly, cancer gene therapy approaches, which are used to regulate specific gene expression in cancer and immune cells, have shown therapeutic potential in preclinical and clinical studies. However, several key issues, including optimum gene transfer, gene transfer vector biology and safety, and long-term persistence in the host, must still be resolved (Husain et al., 2015). Gene transfer is generally achieved with viral and nonviral vectors and nonviral vectors have advantages over viral vectors (Husain et al., 2015). Polymer-based and lipid-based vectors are often used to carry plasmid DNA. Moreover, conjugates on vectors, such as antibodies and ligands, allow plasmid DNA to enter cancer cells (Kaczmarek et al., 2017).

The expression patterns of follicle-stimulating hormone (FSH) receptor (FSHR) in normal and cancer tissues provide an opportunity for drug delivery with high selectivity in ovarian cancer. FSHR is selectively expressed in ovaries, while FSHR expression is low or nonexistent in most noncancerous cells. An analysis based on TCGA datasets has shown that FSHR is expressed in multiple histological subtypes of ovarian cancer (Perales-Puchalt et al., 2017). We have previously reported a targeted delivery system for paclitaxel and RNA interference (RNAi) drugs of growth-regulated oncogene α (gro-α) using FSHR-binding fragments as targeting moieties, which promoted the uptake of drugs by FSHR-expressing ovarian cancer cells (Zhang et al., 2009; Hong et al., 2013; Hong et al., 2018).

However, the use of RNAi drugs is limited not only by the existence of the target genes of RNAi drugs in noncancerous tissues but also by the inevitable off-target effects of RNAi drugs. Therapeutics that directly knockdown agent of interest might result in unpredictable toxic effects in normal tissues. The ideal approach is to target therapeutic genes that are only expressed in cancer cells and not in normal cells. Hence, tumor-specific promoters are used in gene vectors, and these vectors can be used to conditionally regulate therapeutic gene expression in tumor or normal tissues (Dwyer et al., 2005; Lo et al., 2005; Sugio et al., 2011; Nissim et al., 2017). The promoter of mucin 16 (MUC16), also known as CA125, might be a potential tool to drive tumor-localized gene expression in ovarian cancer cells since CA125 is overexpressed in 80% of ovarian carcinomas, and serum CA125 is used as a marker for the monitoring of the treatment response (Bast and Spriggs, 2011).

Here, we screened the possible key sequences of the MUC16 promoter and constructed MUC16 promoter-driven gro-α shRNA plasmids. Then, the plasmids were loaded into a drug delivery system, namely an FSH peptide-conjugated polyethylene glycol (PEG)-polyethylenimine (PEI) copolymer that we previously developed, to target FSHR-expressing ovarian cancer cells. Furthermore, the antitumor effects of the nanoparticle complexes with dual modifications of the MUC16 promoter and FSH peptide were evaluated in ovarian cancer *in vitro* and *in vivo*.

## Materials and methods

### Bioinformatics analysis

To predict the possible key sequences of the MUC16 promoter, the genomic sequences of human MUC16 (GRCh38 genome assembly) were analyzed using online bioinformatics prediction tools, including Neural Network Promoter Prediction, SCOPE, the cis-Regulatory Element Database (cisRED) and the Transcriptional Regulatory Element Database (TRED).

### MUC16 promoter cloning

The predicted promoter sequences were generated from oligonucleotides by PCR. The PCR products were cloned into TA cloning vector pMD18-T (TAKARA Bio Inc., Japan), and the vector was transfected into DH5a competent cells. Then, the TA cloning vector was digested and ligated with the basic vector pGL4.10[*luc2*] (Promega Corporation, USA) at the *Xho*I and *Bgl*II restriction sites. The pGL4.10 vector had no promoter and encodes the luciferase reporter gene *luc2*. The ligated product was named pGL-MUC16. The CMV promoter was also ligated to pGL4.10, and the product was named pGL-CMV and used as the positive control in the experiments described below.

### Luciferase assay

Luciferase reporter gene expression by the MUC16 and CMV promoters was determined in ovarian cancer cells using dual-luciferase reporter assays (Promega Corporation, USA) according to the manufacturer’s protocol. Cells cultured in 24-well plates were transfected with 1.5 µg of plasmids pGL-MUC16 and pGL-CMV for 4 h at 37 °C. pGL-CMV was used as the positive control, and basic pGL4.10 was used as the negative control. The cells were harvested 24 h after transfection. Luciferase assays were then performed, and light units were measured with a microplate reader. The ratio of the Firefly to Renilla luciferase activity was calculated.

### Construction of the plasmid containing gro-α shRNA driven by the MUC16 promoter

The plasmid containing gro-α shRNA driven by the MUC16 promoter was constructed by Invitrogen Corporation, Shanghai, China. The gro-α shRNA plasmid (pcDNA6.2-GW/EmGFP-miR) was screened and cloned in our previous study. The oligonucleotide sequences of gro-α shRNA were as follows: TGCTGTATAATAGGACAGTGTGCAGGGTTTTGGCCACTGACTGACCCTGCACAGTCCTATTATA (sense) and CCTGTATAATAGGACTGTGCAGGGTCAGTCAGTGGCCAAAACCCTGCACACTGTCCTATTATAC (anti-sense). The fragment of MUC16 promoter-EGFP-5’flanking-gro-α shRNA-3’flanking was generated by PCR amplification, spliced from gro-α shRNA plasmid pcDNA6.2 and pGL-MUC16, and then cloned into the pcDNA3.1 + vector at the *Mlu*I and *Nhe*I restriction sites (named MUC16-shGro). The products were transformed into DH5a competent cells, and a single clone was selected, amplified and sequenced.

### Preparation of nanoparticles loaded with theMUC16-shGro plasmid

The pcDNA3.1 + vector containing gro-α shRNA driven by the MUC16 promoter (MUC16-shGro) was loaded into PEG-PEI copolymers. The nanoparticles modified with or without FSH β 33-53 peptide (YTRDLVYKDPARPKIQKTCTF) were prepared as described in our previous study (Hong et al., 2013; Hong et al., 2018). Briefly, the mixture of FSH peptides and Maleimide PEG NHS (MW 3,500 Da) (Jenkem Technology, China) was allowed to react for 6 h at room temperature with a molar ratio of 4:1. FSH peptides and PEG were conjugated using the sulfhydryl groups of peptides and the maleimide of PEG. The product was named FSH-PEG. Then, the mixture of branched PEI (MW 25,000 Da) (Sigma Aldrich Co., St. Louis, MO) and FSH-PEG or PEG was allowed to react for 24 h at room temperature with molar ratio of 1:20 PEG:PEI amine. The products, namely, the PEG-PEI or FSH-PEG-PEI copolymers, were used to condense the plasmid DNA at different molar ratios of nitrogen from PEI to phosphate from the plasmid DNA (N/P).

The condensation of the plasmids by the copolymers was verified by gel retardation analysis. The plasmids and copolymers at N/P ratios of 1, 5, 10, 15, 20, 25 and 30 were mixed with 10 × loading buffer and visualized by 1% agarose gel electrophoresis with ethidium bromide staining. The bands were imaged using a UV imaging system. The conjugation of peptides, PEG and PEI was detected by ^1 ^H nuclear magnetic resonance (NMR) spectroscopy in deuterium oxide. The morphology, particle size and zeta potential were detected using a transmission electron microscope (JEOL Ltd., Japan) and Malvern Zetasizer autosize 4700 (Malvern Instruments Ltd., Malvern, UK).

### Cell culture

Human ovarian cancer cell lines SKOV-3, Caov3, HEY and RMUG-L were archived in our laboratory. SKOV-3 cells were cultured in McCoy’s 5 A Medium, Caov3 cells were cultured in DMEM, and HEY and RMUG-L cells were cultured in RPMI-1640 medium at 37 °C in a 5% CO_2_ environment. The medium was supplemented with 10% fetal bovine serum.

### Immunocytochemistry

After fixation with 4% paraformaldehyde, cells were incubated with MUC16 antibody (Abcam Ltd., HK) at 4 °C overnight. Cells were then washed and incubated with HRP-conjugated secondary antibody (Abcam Ltd., HK) for 30 min. Diaminobenzidine and hematoxylin were used to stain the cells. Images were captured by light microscopy (Olympus Corporation, Tokyo, Japan).

### Enzyme-linked immunosorbent assay

The levels of gro-α secreted by the ovarian cancer cells were determined using a gro-α ELISA kit (R&D Systems Inc., Minneapolis, MN) according to the manufacturer’s instructions. Cell supernatants were collected at 24 h and 48 h after treatment with the nanoparticle complexes. The plasmid concentration was 1.5 μg/μl. Cells were also treated with naked plasmids, and the untreated cells were used as the control. Cell supernatants were added to the 96-well plate coated with human gro-α antibody, and the plate was incubated for 2 h at room temperature. After several washes, the gro-α conjugate was added, and the plate was incubated for 2 h at 4 °C. The color reaction was developed with the substrate solution and blocked with the stop solution. The optical densities were measured at 450 nm.

### Western blot

Gro-α protein expression was determined by Western blot. Ovarian cancer cells were lysed and the protein extracts were separated by SDS-PAGE and blotted onto a polyvinylidene difluoride membrane. The membrane was incubated with gro-α or actin antibody at 4 °C overnight, and then incubated with HRP-conjugated secondary antibody (Abcam Ltd., HK) for 1 h at room temperature. The visualization of the protein bands was performed by a chemiluminescence method and imaged by an ImageQuant™ LAS4000 system (GE Healthcare LifeSciences, Marlborough, MA).

### Cell viability and proliferation

Cell viability and proliferation were determined by CCK-8 analysis (Dojindo Laboratories, Kumamoto, Japan). Cells were seeded in 96-well plates and treated with nanoparticle complexes for 24 h and 48 h. Cells were also treated with MUC16-shGro plasmids, and the untreated cells were used as the control. The plasmid concentration was 1.5 μg/μl. Then, 10 μl of the CCK-8 solution was added and incubated for 1 h at 37 °C. The optical densities were measured at 450 nm using a microplate reader.

### Invasion assay

To study the effect of nanoparticles loading MUC16-shGro plasmid on the invasion capability of cells, 1 × 104 cells after treated with nanoparticle complexes were seeded into the 8.0-μm pore size transwell inserts in serum-free medium. The plasmid concentration was 3 μg/μl. The inserts were pre-coated with 10% Matrigel (BD BioSciences, San Jose, CA). The bottom 24-well plate contained medium supplemented with 10% fetal bovine serum. After incubation for 24 h, the cells remained inside the inserts were gently removed. The cells invaded to the lower surface of the inserts were fixed with 4% paraformaldehyde, stained with hematoxylin and counted under a light microscope.

### Tumor model and antitumor efficacy study

To evaluate the antitumor efficacy of the nanoparticle complexes *in vivo*, nude mice bearing human ovarian cancer HEY xenografts were established. Four- to six-week-old female BALB*/*c nude mice were purchased from and maintained in Shanghai Laboratory Animal Center, Chinese Academy of Sciences. All animal experiments were conducted in accordance with the principles for laboratory animal use and care and with approval from the Ethics Committee for Animal Experimentation. A total of 1 × 10^7^ HEY cells were subcutaneously injected into the flanks of the mice. When the tumors became palpable, the following were administered to the mice via the tail vein at a dose of 5 mg/kg body weight: the nanoparticle complexes conjugated with or without the FSH peptide, the nanoparticle complexes without the MUC16 promoter or the naked plasmid. Saline was also injected as the control. Beginning on day 1, the injections were performed every 4 days for a total of 6 treatments. During the study, body weight was measured to monitor potential toxic effects. Tumor growth was measured with calipers, and the tumor volume was calculated as length × width^2^/2.

### Statistical analysis

Statistical comparisons were performed with GraphPad Prism 6.0 (La Jolla, CA) and SPSS 16.0 (SPSS Inc., USA). One-way ANOVA was used to compare the data. A *p* value  .05 was considered significant, and the data are shown as the mean ± SD.

## Results

### Screen of MUC16 promoter sequences

Four possible promoter sequences were predicted in regions that were 4 kbp upstream of MUC16 using online bioinformatics prediction tools. The human reference genome assembly was GRCh38. The positions of the four sequences were as follows: chr19:8981710-8981777 (TAAA repeats) (named MUC16.1), chr19:8981342-8981533 (named MUC16.2), chr19:8981918-8982312 (CT repeats) (named MUC16.3), and chr19:8983940-8984306 (named MUC16.4).

**Figure 1.**
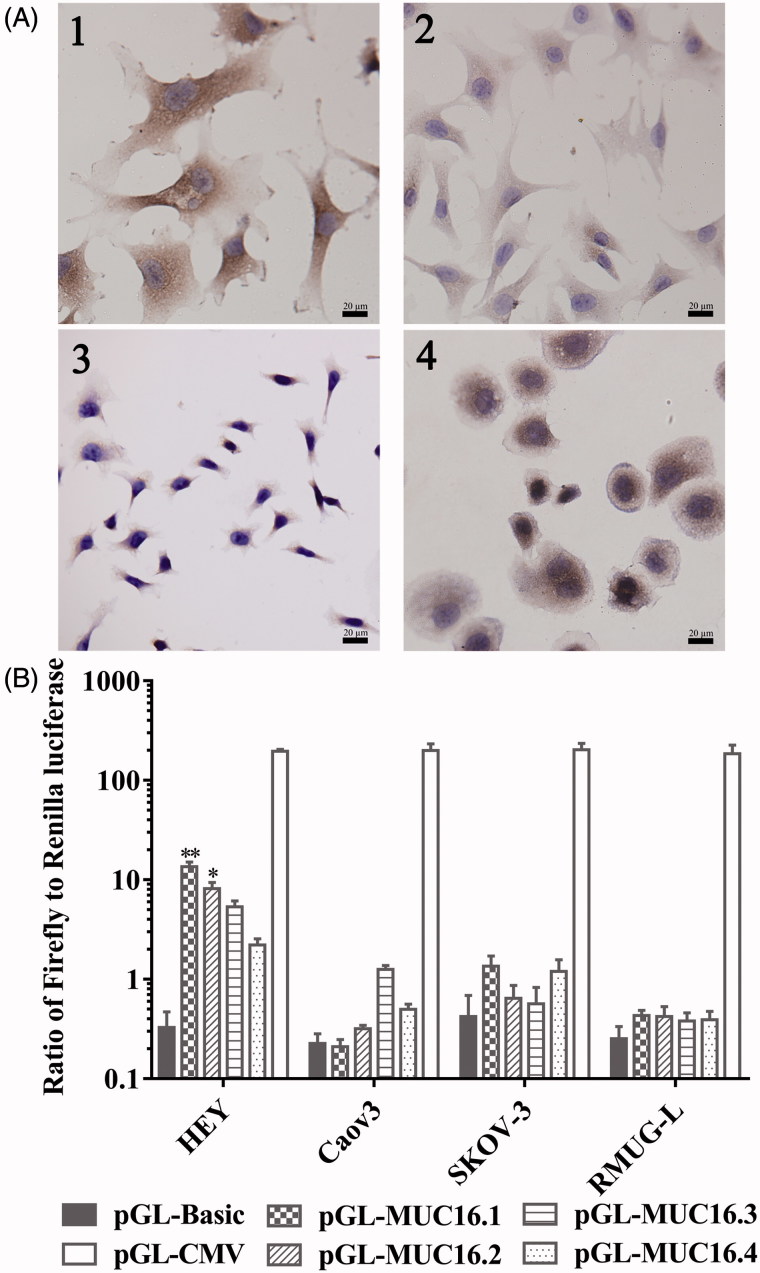
MUC16 expression and transcriptional activity of MUC16 promoters. (A) MUC16 expression in ovarian cancer cells by immunocytochemistry. 1, HEY; 2, Caov3; 3, SKOV-3; 4, RMUG-L. (B) Transcriptional activity of the MUC16 promoters in ovarian cancer cells per dual-luciferase reporter assays. Cells were transfected with pGL4.10 vectors containing different MUC16 promoters (pGL-MUC16.1, pGL-MUC16.2, pGL-MUC16.3 and pGL-MUC16.4). The pGL4.10 vector with the CMV promoter (pGL-CMV) was used as the positive control. pGL4.10 without a promoter (pGL-Basic) was used as the negative control. *P < .05, **P < .01 vs. pGL-Basic.

To investigate the transcriptional activity of the MUC16 promoters, ovarian cancer cells were transfected with the pGL4.10 vector containing different MUC16 promoter sequences, and then, transcriptional activity was measured with dual-luciferase reporter assays. As shown in Figure 1, the MUC16.1 and MUC16.2 promoter constructs resulted in significantly higher luciferase gene expression in HEY cells with positive MUC16 than the control, but MUC16.3 and MUC16.4 promoter constructs did not result in a significant increase in luciferase gene expression. All of the four sequences did not exhibit the significantly increased luciferase gene expression in Caov3, SKOV-3 and RMUG-L cells with low or negative basal MUC16 levels. These data indicated that the MUC16.1 and MUC16.2 promoters could result in the target gene expression. The MUC16.1 and MUC16.2 sequences were used in subsequent experiments.

### Preparation and characterization of the nanoparticles loaded with MUC16-shGro plasmid

To drive gro-α shRNA expression, a fragment of MUC16 promoter-gro-α shRNA was generated by PCR, and then cloned into the pcDNA3.1 + vector at the *Mlu*I and *Nhe*I restriction sites. The MUC16.1 and MUC16.2 promoter sequences with high transcriptional activity were used. The cut position of *Mlu*I was base 229, and that of *Nhe*I was base 896. The CMV promoter of pcDNA3.1 + was located within bases 232-819. Thus, the CMV promoter was replaced with the MUC16 promoter by restriction enzyme digestion, and the product was confirmed by sequencing.

**Figure 2.**
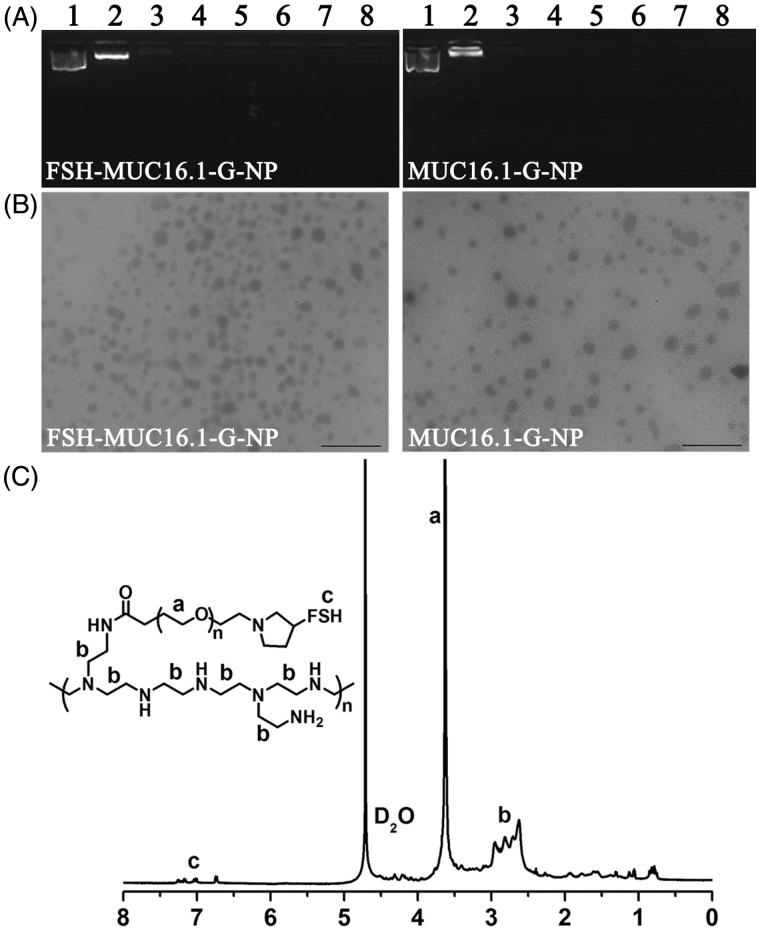
Characterization of nanoparticle complexes. (A) The condensation of plasmids by the PEG-PEI copolymers as determined by gel retardation analysis. Lane 1, naked plasmid; lanes 2 to 8, nanoparticle complexes at N/P ratios of 1, 5, 10, 15, 20, 25 and 30, respectively. (B) Transmission electron micrograph of the nanoparticle complexes. Bar, 1 μm. (C) The ^1^H NMR spectra of the FSH-PEG-PEI copolymer. The peaks at 4.6–4.7 ppm were solvent D_2_O. The FSH peptides, PEG (-OCH2CH2-) and PEI (-CH2CH2NH-) were represented by the peaks at 7.0–7.2 ppm, 3.5-3.6 ppm and 2.4–3.0 ppm.

Then, the plasmids were loaded into PEG-PEI copolymers modified with (named FSH-MUC16.1-G-NP and FSH-MUC16.2-G-NP) or without the FSH β 33-53 peptide (named MUC16.1-G-NP and MUC16.2-G-NP). As shown in Figure 2(A,B), the negative-charge plasmids were neutralized and retarded by the positive-charge PEG-PEI copolymers in the gel. When the N/P ratio was greater than 5, the plasmids were completely condensed by the PEG-PEI copolymers. A N/P ratio of 25 was used in subsequent experiments according to our previous study. The ^1^H NMR spectra of the FSH-PEG-PEI copolymer exhibited the peaks from the FSH peptides (7.0-7.2 ppm), -OCH2CH2- of PEG (3.5-3.6 ppm) and -CH2CH2NH- of PEI (2.4-3.0 ppm), which suggested that the FSH peptides, PEG and PEI were successfully conjugated (Figure 2(C)). The particle size values of FSH-MUC16.1-G-NP and FSH-MUC16.2-G-NP were 196-223 nm, and those of MUC16.1-G-NP and MUC16.2-G-NP were 158-181 nm. The zeta potential values of FSH-MUC16.1-G-NP and FSH-MUC16.2-G-NP were 29–37 mV, and those of MUC16.1-G-NP and MUC16.2-G-NP were 36–46 mV.

### Nanoparticles loaded with MUC16-shGro plasmid decreased gro-α expression

**Figure 3.**
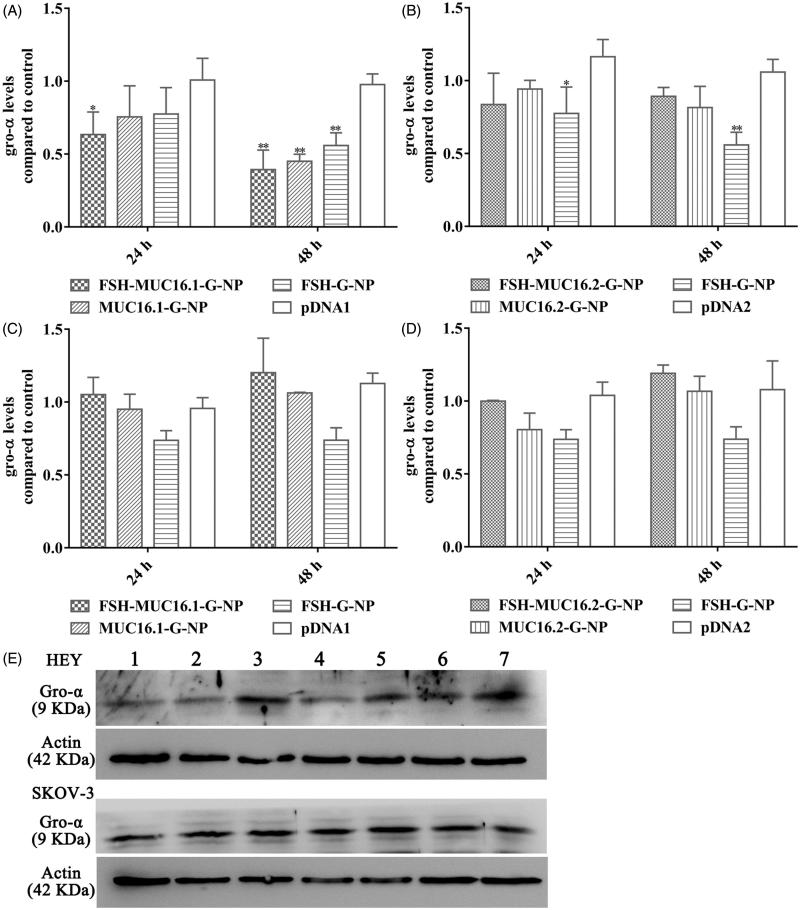
The levels of gro-α expression in ovarian cancer cells treated with the nanoparticle complexes by ELISA and Western blot. HEY cells treated with the nanoparticle complexes containing the MUC16.1 (A) or MUC16.2 (B) promoter. SKOV-3 cells treated with the nanoparticle complexes containing the MUC16.1 (C) or MUC16.2 (D) promoter. Cells were treated with FSH-MUC16.1-G-NP, MUC16.1-G-NP, FSH-G-NP, FSH-MUC16.2-G-NP, MUC16.2-G-NP or naked plasmid containing gro-α shRNA driven by the MUC16.1 (pDNA1) or MUC16.2 (pDNA2) promoter. *p < .05, **p < .01 versus pDNA1 or pDNA2. (E) Gro-α protein expression was determined by Western blot. Lanes 1, 2, 3, 4, 5, 6 and 7, FSH-MUC16.1-G-NP, MUC16.1-G-NP, pDNA1, FSH-G-NP, FSH-MUC16.2-G-NP, MUC16.2-G-NP and blank control.

To investigate the silencing effects of the nanoparticle complexes on the target gene, the levels of gro-α secreted in supernatants of the ovarian cancer cells were measured by ELISA. FSHR expression in the ovarian cancer cells that we used has been reported previously (Hong et al., 2013; Hong et al., 2018). As shown in Figure 3, HEY cells (which are positive for both FSHR and MUC16) exhibited a decrease in gro-α secretion levels at 24 h and 48 h after treatment with the nanoparticle complexes containing the MUC16.1 promoter. The levels of gro-α were reduced to 63.4% and 39.3% at 24 h and 48 h, respectively, relative to the control level after treatment with the FSH-MUC16.1-G-NP, MUC16.1 promoter-driven and FSH peptide-conjugated nanoparticle complexes. However, the nanoparticle complexes containing the MUC16.2 promoter did not significantly reduce the gro-α levels in HEY cells. In SKOV-3 cells (which exhibit low or negative FSHR and MUC16 expression), neither the MUC16.1 nor MUC16.2 promoter-driven nanoparticle complexes showed significant inhibition on the gro-α secretion levels. Additionally, FSH peptide-conjugated nanoparticles containing gro-α shRNA pcDNA6.2 plasmid (FSH-G-NP) that had a CMV promoter significantly decreased gro-α secretion levels in HEY cells, but no significant decrease was observed in SKOV-3 cells, which was similar to our previous study. To further confirm the ELISA result, we used Western blot to detect gro-α protein expression in the ovarian cancer cells at 48 h after treatment. The reduction of gro-α expression was observed in HEY cells treated with FSH-MUC16.1-G-NP, MUC16.1-G-NP and FSH-G-NP (Figure 3(E)). These data indicated that the MUC16.1 promoter-driven nanoparticle complexes could effectively decrease gro-α secretion, along with FSH peptide modification, which was used in subsequent experiments.

### Nanoparticles loaded with the MUC16-shGro plasmid inhibited the proliferation and invasion of ovarian cancer cells

**Figure 4.**
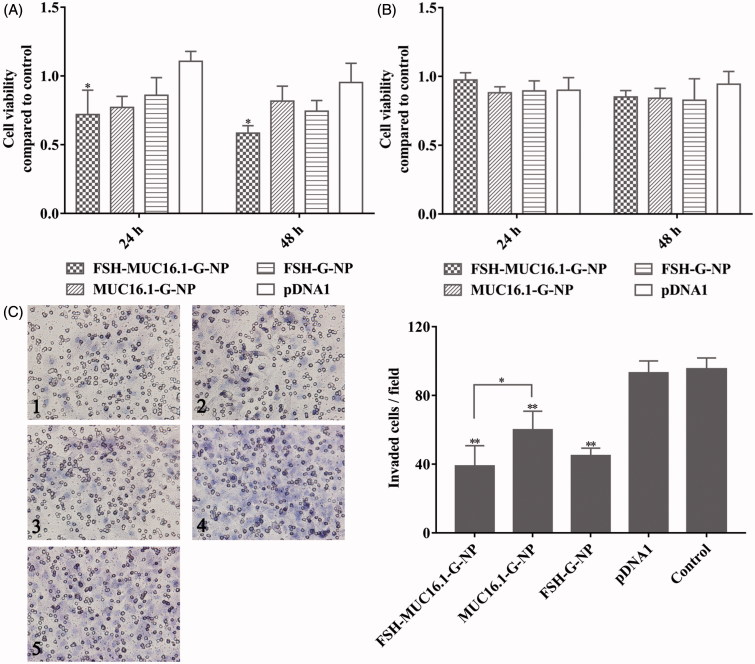
Viability and invasion of ovarian cancer cells treated with the nanoparticle complexes per CCK-8 and transwell assays. HEY cells (A) and SKOV-3 cells (B) were treated with FSH-MUC16.1-G-NP, MUC16.1-G-NP, FSH-G-NP or pDNA1 for 24 h and 48 h. Untreated cells were used as the control. *p < .05, **p < .01 versus pDNA1. (C) Cell invasion as determined by transwell assay in HEY cells treated with different nanoparticle complexes for 24 h. 1, FSH-MUC16.1-G-NP; 2, MUC16.1-G-NP; 3, FSH-G-NP; 4, pDNA1; 5, blank control. *p < .05, **p < .01 versus. pDNA1 or control.

As described above, the MUC16.1 promoter-driven nanoparticle complexes decreased gro-α secretion in ovarian cancer cells. A previous study has reported that gro-α overexpression promotes the senescence of fibroblasts and ovarian tumorigenesis (Yang et al., 2006). Thus, ovarian cancer cell proliferation and invasion after treatment with the nanoparticle complexes was measured in this study. As shown in Figure 4(A,B), HEY cell proliferation was greatly inhibited by the MUC16.1 promoter-driven nanoparticle complexes, while SKOV-3 cell proliferation was not inhibited effectively. The viability of HEY cells treated with FSH-MUC16.1-G-NP was 71.9% and 58.4% at 24 h and 48 h, respectively. We next investigated ovarian cancer cell invasion using transwell assays. The plasmid concentration was increased by 2-fold compared with the concentration in the cell viability experiment. As shown in Figure 4(C), significantly decreased cell invasion was observed in HEY cells treated with FSH-MUC16.1-G-NP, MUC16.1-G-NP and FSH-G-NP compared to HEY cells treated with pDNA1 or control. The suppression by FSH-MUC16.1-G-NP was more effective than the suppression by the complexes without the FSH peptide in the FSHR-positive HEY cells. The effects of FSH peptide-conjugated nanoparticles containing gro-α shRNA (FSH-G-NP) were similar to those observed in our previous study. Together, these data supported that the dual modifications of the MUC16.1 promoter and FSH peptide on nanoparticles containing gro-α shRNA could enhance inhibitory effects of gro-α shRNA on ovarian cancer cells.

### Nanoparticles loaded with the MUC16-shGro plasmid suppressed tumor growth in vivo

**Figure 5.**
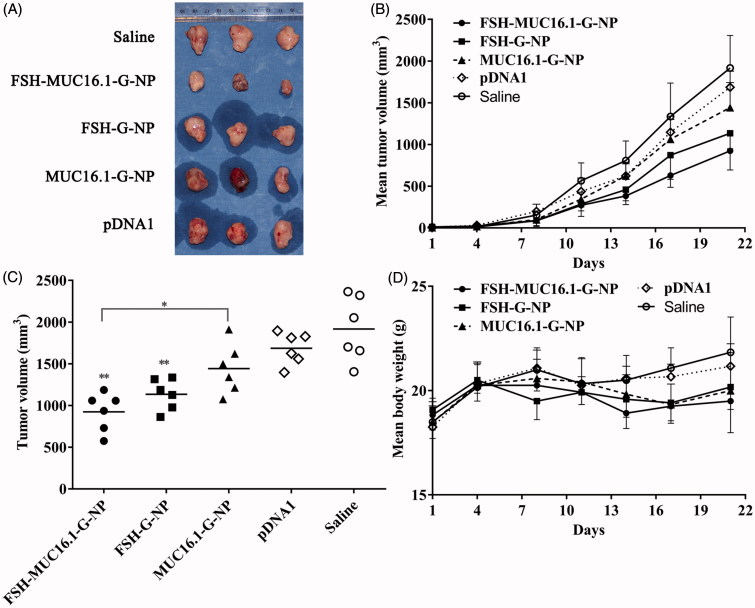
The antitumor efficacy in a nude mouse model bearing human ovarian cancer HEY xenografts. (A) The morphology of the tumor xenografts. (B) The mean tumor growth curves. (C) The tumor volumes at the study endpoint. *p < .05, **p < .01 versus pDNA1 or saline. (D) The body weight changes of the mice. The mice were intravenously injected with FSH-MUC16.1-G-NP, MUC16.1-G-NP, FSH-G-NP, naked plasmid (pDNA1) or saline.

The antitumor efficacy of nanoparticle complexes was evaluated in a nude mouse model bearing human ovarian cancer HEY xenografts. The mice were intravenously injected with FSH-MUC16.1-G-NP, MUC16.1-G-NP, FSH-G-NP, naked MUC16.1-shGro plasmid or saline. Tumor growth was observed for 3 weeks. As shown in Figure 5, the tumors continuously grew in the mice treated with saline. The naked plasmid treatment resulted in a slight regression of the tumor volumes. In contrast, tumor growth was significantly suppressed in the mice that received FSH-MUC16.1-G-NP and FSH-G-NP compared with naked plasmid or saline. FSH-MUC16.1-G-NP exhibited significantly enhanced tumor regression compared with MUC16.1-G-NP. At the study end point, the average tumor growth inhibition levels of FSH-MUC16.1-G-NP, MUC16.1-G-NP and FSH-G-NP were 51.8%, 24.7% and 40.8%, respectively. The tumor regression level of FSH-G-NP was consistent with that of our previous study. FSH-G-NP exhibited more effective tumor inhibition *in vivo* than MUC16.1-G-NP, which might be due to FSH-G-NP contained a CMV promoter as well as FSH peptide-mediated active targeting increased the plasmid uptake. These data indicated that the dual modifications of the MUC16.1 promoter and FSH peptide on the nanoparticles containing gro-α shRNA enhanced the *in vivo* antitumor efficacy. Although the mice in the FSH-MUC16.1-G-NP treatment group experienced a body weight loss of approximately 10% compared to the saline group, no significant differences were evident among the groups.

## Discussion

In this study, we developed a MUC16 promoter-driven shRNA expression vector for tumor-localized gene expression in ovarian cancer. This vector was specifically delivered into FSHR-expressing ovarian cancer cells through FSH peptide-conjugated nanoparticles. Our strategy was dual targeting of ovarian cancer via a MUC16 promoter trigger and FSHR mediation, which could enhance therapeutic specificity by avoiding the expression of passive-uptake shRNA drugs in non-cancer cells and which could reduce off-target effects.

A safe and effective vector is vital for the clinical use of cancer gene therapy. Nonviral vectors, including polymers and lipids, have been the focus of relevant investigations because these vectors are associated with a high delivery efficiency. To minimize toxicity in noncancerous tissues, tumor-specific ligands are used to modify vectors to increase the selectivity of the delivery of drugs, such as folate and LHRH analogs (Ledermann et al., 2015; Tambe et al., 2017). A receptor-ligand-mediated drug delivery strategy can efficiently deliver therapeutic genes.

In our studies, FSHR-binding peptides were conjugated to the surfaces of PEG-polylactic acid (PLA) and PEG-PEI nanoparticles as targeting moieties because of the limited expression of FSHR in the body (Zhang et al., 2009; Hong et al., 2013). The FSH peptides enabled the selective uptake of paclitaxel or a therapeutic gene by ovarian cancer cells. The nanoparticles loaded with paclitaxel exhibited great antitumor effects, even with a small dose of paclitaxel. When gro-α siRNA or shRNA was delivered into ovarian cancer cells through this carrier, antitumor effects were observed, and these effects were based on gro-α involvement in the development and progression of ovarian cancer. Thus, the FSHR-targeted strategy was applied in this study. After the MUC16 promoter was introduced into the FSH peptide-conjugated gro-α shRNA nanoparticles, tumor growth was significantly inhibited in a nude mouse model bearing ovarian cancer, and no obvious toxic effects were observed.

Ideal gene therapy involves tumor-specific killing that can be achieved by delivering therapeutic genes under the control of tumor-specific promoters into cancer cells (Lo et al., 2005; Rama et al., 2015). For example, the survivin, telomerase reverse transcriptase, CEA, AFP and Her-2 promoters show good transcriptional control and can specifically drive the expression of therapeutic genes (Lo et al., 2005; Zhang et al., 2009; Garg et al., 2010; Kwon et al., 2010; Machitani et al., 2017). The mucin family consists of large glycoproteins that are produced by epithelial cells, and mucin expression has been suggested to be tissue- and cell-type specific (Yamada et al., 2011). Interestingly, mucins are often overexpressed and abnormally glycosylated in many carcinomas (Yamada et al., 2011). Thus, the promoters of mucin genes, such as mucin 1 (MUC1), have been used to drive the expression of genes of interest in cancer cells. The MUC1 promoter exhibits high transcriptional activity and can drive the expression of genes of interest in adenovirus or plasmid vectors for breast cancer targeted therapy (Gao et al., 2007; Trujillo et al., 2009; Doloff et al., 2011; Tholey et al., 2015).

MUC16/CA125, a member of the mucin family, is a large transmembrane glycoprotein with tandem repeat regions and with enriched O-linked and N-linked glycosylation (O’Brien et al., 2001; Bouanene & Miled, 2010). CA125 is overexpressed in ovarian carcinoma and is a well-known tumor biomarker, especially for ovarian cancer. In addition, MUC16 is involved in ovarian cancer development and has been applied in targeted therapy against ovarian cancer, including CA125 antibodies (Felder et al., 2014). And an interesting phenomenon was observed in this study, as a transmembrane mucin, MUC16 also expressed in the cytoplasm of ovarian cancer cell lines. Data from an immunohistochemistry analysis in 114 pancreatic ductal adenocarcinoma tissue samples show that MUC16 cytoplasmic expression elevates with increasing cancer stage, and is an independent predictor of poor prognosis (Higashi et al., 2015). The location in cells might be related to MUC16 cleavage. The cleavage site of MUC16 locates in the juxta-membrane ectodomain. The cleaved N-terminal fragment is secreted from the cell and generates circulating CA125. The cleaved carboxyl-terminal fragment remains associated with the cells and is found to be localized into the nucleus (Das & Batra, 2015). However, the relationship between the MUC16 cytoplasmic or membranal expression and ovarian cancer development remains unclear. The abnormal glycosylation of CA125 has shown high potential for ovarian cancer diagnosis in preclinical studies (Qian et al., 2013). Site-specific N-linked glycosylation of the MUC16 ectodomain mediates cell surface interactions, and monoclonal antibodies directed at the ectodomain N-linked glycosylation sites can block ovarian cancer growth (Rao et al., 2017). The implications of MUC16 in ovarian cancer provide optimal design strategies for cancer targeted therapy.

Despite the good understanding of the protein structure and function, the transcriptional regulation of the MUC16 gene remains unclear. MUC16 was cloned and mapped to a mucin cluster on chromosome 19p13 (O'Brien et al., 2001; Yin & Lloyd, 2001). CA125 mRNA is approximately 66,765 bp and encodes a theoretical product that is 22,152 amino acids in length (O'Brien et al., 2001; Bouanene & Miled, 2010). Epigenetic regulation has been suggested to be involved in MUC16 expression. However, DNA methylation and histone H3-K9 modifications in the 5’ flanking region of MUC16 are unlikely to regulate MUC16 transcription (Yamada et al., 2011). The start site of MUC16 transcription has been determined by 5′ Rapid amplification of cDNA ends (RACE) in ovarian cancer cell lines, and NFκB binding site is found in the region within 200 bp from the start of transcription (Morgado et al., 2016). Putative c-Myc binding E-box elements are found in the -2500 to 200 bp of the transcription start site in MUC16 gene (Liang et al., 2017).

Here, we predicted the promoter sequences in the regions that were 4 kbp upstream of MUC16 and measured transcriptional activity in ovarian cancer cells with dual-luciferase reporter assay. As shown in our study, although the MUC16.1 and MUC16.2 promoter constructs resulted in higher luciferase gene expression in HEY cells, the nanoparticle complexes containing the MUC16.2 promoter-driven plasmid did not significantly reduce the gro-α levels. It seemed that the promoter sequence with TAAA repeats, the MUC16.1 promoter, showed high transcriptional activity, and this sequence might contain an atypical TATA box and be a transcriptional recognition site. The plasmid containing gro-α shRNA driven by this MUC16 promoter sequence decreased gro-α protein secretion in ovarian cancer cells. However, the MUC16.2 promoter sequence had no possible transcriptional recognition sites such as TATA box. The MUC16.3 promoter sequence with CT repeats that made up a potential cis-acting element did not exhibit transcriptional activity. However, the transcriptional activity of the MUC16 promoter that we screened was lower than that of the positive control, namely, the CMV promoter. Some DNA elements, including enhancers, might need to be integrated into vectors to increase transcriptional activity in future studies (Rama et al., 2015).

## Conclusions

Our study provides a novel gene delivery system using a MUC16 promoter trigger and FSH peptide-mediated active targeting for ovarian cancer. Although further evaluations are needed through the experiments of primary ovarian cancer cells or peritoneal mesothelial cells, the approach we designed may be a promising strategy and may be applied in various cancers for specific genetic therapeutic delivery.
